# Variation in the psychosocial determinants of the intention to prescribe hormone therapy prior to the release of the Women's Health Initiative trial: a survey of general practitioners and gynaecologists in France and Quebec

**DOI:** 10.1186/1472-6947-5-31

**Published:** 2005-09-08

**Authors:** France Legare, Gaston Godin, Virginie Ringa, Sylvie Dodin, Lucile Turcot, Joanna Norton

**Affiliations:** 1 CHUQ, St-François d'Assise Hospital Research Center, 10 rue de l'Espinay, Quebec, QC, Canada, G1L 3L5; 2 Canada Research Chair on Behaviour and Health, Faculty of Nursing, Laval University, Quebec, QC, Canada, G1K 7P4; 3 INSERM National Institute for Medical Research U149, Epidemiological Research Unit on Perinatal Health and Women's Health, 16, ave Paul Vaillant Couturier, 94807 Villejuif cedex, France

## Abstract

**Background:**

Theory-based approaches are advocated to improve our understanding of prescription behaviour. This study is an application of the theory of planned behaviour (TPB) with additional variables. It was designed to assess which variables were associated with the intention to prescribe hormone therapy (HT). In addition, variations in the measures across medical specialities (GPs and gynaecologists) and across countries (France and Quebec) were investigated.

**Methods:**

A survey among 2,000 doctors from France and 1,044 doctors from Quebec was conducted. Data were collected by means of a self-administered questionnaire. A clinical vignette was used to elicit doctors' opinions. The following TPB variables were assessed: attitude, subjective norm, perceived behavioural control, attitudinal beliefs, normative beliefs and power of control beliefs. Additional variables (role belief, moral norm and practice pattern-related factors) were also assessed. A stepwise logistic regression was used to assess which variables were associated with the intention to prescribe HT. GPs and gynaecologists were compared to each other within countries and the two countries were compared within the specialties.

**Results:**

Overall, 1,085 doctors from France returned their questionnaire and 516 doctors from Quebec (response rate = 54% and 49%, respectively). In the overall regression model, power of control beliefs, moral norm and role belief were significantly associated with intention (all at *p *< 0.0001). The models by specialty and country were: for GPs in Quebec, power of control beliefs (*p *< 0.0001), moral norm (*p *< 0.01) and cytology and hormonal dosage (both at *p *< 0.05); for GPs in France, power of control beliefs and role belief (both at *p *< 0.0001) and perception of behavioural control (*p *< 0.05) and cessation of menses (*p *< 0.01); for gynaecologists in Quebec, moral norm and power of control beliefs (both at *p *= 0.01); and for gynaecologists in France, power of control beliefs (p < 0.0001), and moral norm, role belief and lipid profile (all at *p *< 0.05).

**Conclusion:**

In both countries, compared with GPs, intention to prescribe HT was higher for gynaecologists. Psychosocial determinants of doctors' intention to prescribe HT varied according to the specialty and the country thus, suggesting an influence of contextual factors on these determinants.

## Background

Until the results of the Women's Health Initiative trial [1] were known, hormone therapy (HT) was promoted for preventing osteoporosis and was thought to protect from cardiovascular diseases [2]. Although physicians agreed that menopause was not a disease, they considered it a serious health problem [3]. Hot flashes, night sweats and osteoporosis were reasons for gynaecologists and general practitioners (GPs) to prescribe HT [4]. Notwithstanding these benefits, breast cancer was a major concern [5,6]. Moreover, many women objected to adopting HT at menopause from fear of medicalization of an otherwise natural process [7]. Regardless of these conflicting evidences, in Canada, at the time this study was conducted, Premarin^®^, a known estrogenic compound, was the most prescribed drug by gynaecologists and the third most prescribed drug by GPs [8].

Following the Women's Health Initiative trial [1], there was a decrease in the number of prescriptions of standard-dose HT but a slight increase in the number of prescriptions of low-dose HT in the United States [9]. In fact, HT remains the most effective option to alleviate severe perimenopausal symptoms such as hot flashes [10]. Moreover, although HT is not a first-line treatment recommended for preventing osteoporosis [11], its use is associated with a decrease in fractures in current users [12]. Based on the results of the Women's Health Initiative trial, HT use for 1 year in 10 000 healthy postmenopausal women is associated with 7 more cardiovascular disease events, 8 more invasive breast cancers, 8 more strokes, 8 more pulmonary emboli, 6 fewer colorectal cancers and 5 fewer hip fractures [1]. Weighing the benefits with the risks associated with HT reminds women and their doctor that deciding about HT requires a careful and individualised assessment of their personal situation.

It is in this context that decisions about HT is said to be representative of clinical decision-making in the face of scientific uncertainty [13,14]. Thus, it is likely that many factors play a role in a doctor decision-making process leading to a prescription of HT. Attitude towards HT is associated with the medical specialty [15,16], and age [17] as well as gender of the provider [15,18]. The country in which a doctor practices medicine also appeared to play a role [19].

### Conceptual underpinnings of this study

In studies of doctors' decision-making and related prescription behaviours, more attention needs to be given to the use and combination of different theories [20]. The lack of use of theories restrains effective implementation of change in patients' care because it restrains our understanding of the pathways through which a given implementation strategy is effective. The theory of planned behaviour [21] is well known through its previous applications to the study of doctors' behaviours [22-25]. This theory provides a theoretical account of the way in which attitude, subjective norm and perceived behavioural control combine to predict a given behavioural intention (decision-making) [26] and in turn, a given behaviour [27]. Thus, it provides direction for elaborating effective strategies that will influence the decision-making process leading to change in doctors' behaviours.

This theory postulates that under a controlled situation, intention is the immediate determinant of behaviour [21]. In turn, this intention is under the influence of three main factors: attitude, subjective norm and perceived behavioural control. Each can be assessed directly or indirectly (beliefs-based measure). Attitude is conceptualized as a personal evaluation of the action. It is the product of a set of salient beliefs about the consequences of performing the behaviour, each weighted by an evaluation of the importance of the respective consequences. Subjective norm refers to a perceived social pressure to perform the behaviour in question. The belief-based measure requires that the individual's normative beliefs be multiplied by the individual's motivation to comply with these socially-normative referents. Perceived behavioural control is a measure of the amount of control the individual has over the behaviour in question. It refers to the individual's perception of barriers or facilitating factors likely to influence the adoption of the behaviour. It can also be measured directly or indirectly. For the indirect measurements, the individual's control beliefs must be weighted by the corresponding perceived evaluation of how much each of these control beliefs will impact on the adoption of the behaviour. According to the authors of this theory, sociodemographics and other variables will influence behaviour through their influence on the attitude, the subjective norm and the perceived behavioural control [21]. Successful behavioural change will occur only if the underlying determinants of intention change.

**Figure 1:**
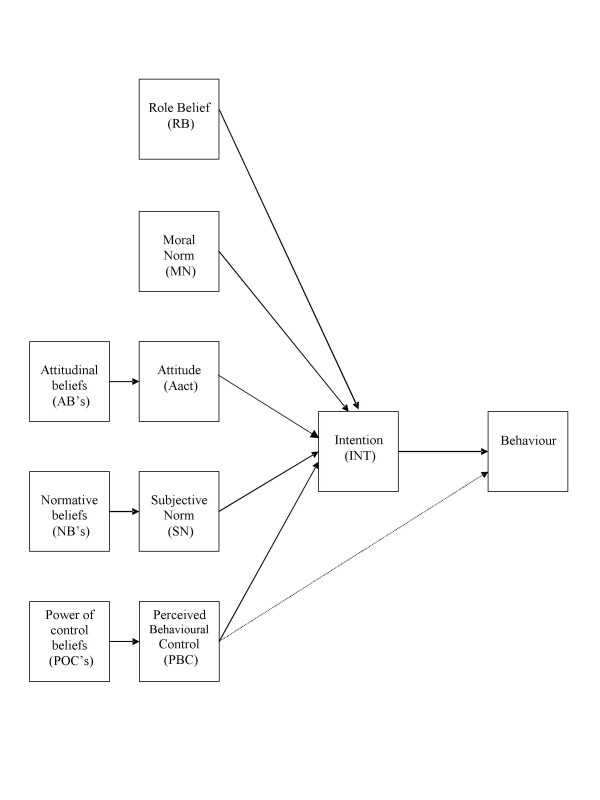
Initial theoretical model.

Although the theory of planned behaviour has proven useful when studying health related behaviours, two systematic reviews found that components of this theory explain on average 41% of the variance in intention and 28% to 31% of the variance in behaviour thus, suggesting that other variables must play a direct role on the behavioural intention and possibly, on the behaviour itself [28,29]. Consequently, some authors have expanded the theory of planned behaviour to include other relevant psychosocial constructs [28]. For example, the measure of moral norm [30] was found to be useful in understanding women's intention to use HT [31,32]. Moral norm takes into account feelings of personal responsibility regarding adoption of a specific behaviour, that is, the individual's perception of the moral correctness or incorrectness of performing the behaviour. Because of the potential influence of the medical specialty, in this study, role belief was also taken into consideration [30]. Role belief refers to the perception by the individual that members of a specific group would perform the behaviour under study. The initial theoretical model adopted as a basis for examining the psychosocial determinants associated with doctors' intention to prescribe HT is presented in Figure 1.

To our knowledge, at the time this study was planned, we were not aware of any studies that had applied the theory of planned behaviour to study variations of doctors' intention to prescribe HT across countries. Physicians in France and the province of Quebec, Canada, share the same language (French) but use it in very different geographical and cultural settings. Therefore, studies of their intention to prescribe HT offered a unique opportunity to provide more insight in the cultural variation in the psychosocial determinants associated with this behaviour without the difficulties associated with having questionnaires in two different languages. Therefore, this study is an application of the theory of planned behaviour with additional variables. It was designed to assess which variables were associated with the intention to prescribe HT. Hypotheses refer mainly to the assumption underlying the theoretical framework. It could nonetheless be added that we hypothesized that variations in the intention to prescribe HT between gynaecologists and GPs would be observed as well as variations in the intention to prescribe HT between French and French Canadians physicians would be observed.

## Methods

### Population and sampling strategy

The study was conducted in 1997 in France and in the province of Quebec, Canada, where French is the prominent language. In France, questionnaires were mailed to a representative sample of 1,000 GPs and 1,000 gynaecologists, randomly drawn from an exhaustive list of 65,000 GPs and 7,000 gynaecologists. This daily updated list was provided by Fournier Pharma, a French pharmaceutical company that is known to have one of the most exhaustive lists of physicians. This list is used for visiting physicians in France. In Quebec, questionnaires were mailed to all gynaecologists (n = 244) and to a representative sample of 800 GPs from an estimated pool of 7000 GPs. This second list was provided by the medical licensing body of this Canadian province.

### Data collection procedure and development of the questionnaire

Data were collected by means of a mailed self-administered questionnaire using a modified Dillman strategy [33]. The development of the questionnaire was performed in both France and Quebec to provide only one questionnaire. The self-administered questionnaire comprised two sections. The first section addressed sociodemographics as well as self-reported practice patterns in the field of menopausal health. The second section assessed the behavioural intention of physician to prescribe HT when faced with a clinical vignette. This clinical vignette was developed in a series of two iterative consultations and tested with a total of 22 GPs and 22 gynaecologists in both countries. It presented a 55 years old menopausal woman who had been menopausal for the past three years. She had no specific opinion about HT and did not complain about hot flashes. This woman had no contra-indication to HT. She had no known risk factors for cardiovascular disease or for osteoporosis. She had a 5-year risk of breast cancer of 2% because her maternal grandmother and her own mother suffered from breast cancer (average risk = 1.5%) [34]. This clinical vignette was purposely designed to maximize the variance in the intention of physicians to prescribe HT.

### Measures

#### Direct measures

In line with the theory of planned behaviour, the behaviour under study was defined as followed: to prescribe (action) HT (target) to a menopausal woman who is consulting for a routine periodical medical exam and who is presented in the clinical vignette (context) [21]. The time frame was not specified. Intention to prescribe HT was assessed by means of two items. After the general comment "If you were the physician of Mrs. X, what would you do?", physicians were asked to answer the following questions on a bipolar 7-point scale: "My intention would be to prescribe her HT." ('unlikely' to 'likely'); "I would prescribe her HT." ('disagreeing' to 'agreeing'). The mean of the composite score was computed (Pearson r = 0.84). Attitude was assessed by means of four items using a semantic differential bipolar 7-point scale. The four pairs of adjectives used were: "not gratifying/gratifying", "not satisfying/satisfying", harmful/harmless" and "not useful/useful". Each pair of adjectives appeared after the sentence: "For me, prescribing HT for Mrs. X would be ...". The mean composite score of the four items was taken as the attitude value (Cronbach α = 0.90). Subjective norm was assessed by means of two items, each assessed on a bipolar 7-point scale. Physicians were invited to indicate their level of agreement with the following statements: "Most of the persons who are important for me in the profession would recommend that I prescribe HT to Mrs. X ", ('disagree' to 'agree'); "The proportion of my colleagues who would prescribe HT to Mrs. X is ..." ('low' to 'high'). These two items were used to compute a mean composite score (Pearson r = 0.84). Two items were included to assess perceived behavioural control, each on a bipolar 7-point response scale. The items were: "I see no barriers to prescribing HT to Mrs. X " ('disagree' to 'agree'); "For me, prescribing HT to Mrs. X would be..." ('difficult' to 'easy'). These two items were used to compute a mean composite score (Pearson r = 0.79). Moral norm was obtained by means of three items, each on a bipolar 7-point response scale: "Given my personal convictions, I would prescribe HT to Mrs. X" ('disagree' to 'agree'); "If I were to prescribe HT to Mrs. X, I would feel guilty" ('agree' to 'disagree'); and "I think that this is totally ethical that I prescribe HT to Mrs. X" ('disagree' to 'agree'). A mean composite score for moral norm was computed (Cronbach α = 0.87). Role belief was obtained by means of two items, each on a bipolar 7-point response scale: "In a situation like the one of Mrs. X, I think that a physician who has the same education as me would prescribe HT" ('disagree' to 'agree'); and "In a situation like the one of Mrs. X, I think that a physician of the same gender as me would prescribe HT" ('disagree' to 'agree'). The mean composite score reliability value for moral norm was 0.87 (Pearson r).

#### Indirect measures

In line with methodological developments in the use of the theory of planned behaviour, only one arm of each indirect measure of the main constructs (attitude, social norm and perception of control) was assessed, that is attitudinal beliefs, normative beliefs and power of control beliefs [35]. Therefore, no multiplicative procedure was applied [36]. Attitudinal beliefs were assessed by means of six items, each on a bipolar 7-point response scale ('disagree' to 'agree'). Following the statement, "If I were to prescribe HT to Mrs. X", physicians were asked to answer if HT would: "reduce bone mass loss", "protect from cardiovascular diseases", "not raise significantly her risk of breast cancer", "reduce completely her hot flushes", "prevent ageing of the urogenital tract" and "improve her breast exam follow-up". These six items were used to compute a mean composite score for attitudinal beliefs (Cronbach α = 0.74). Normative beliefs were assessed with six items, each on a bipolar 7-point response scale ('would not approve' to 'would approve'). Individuals or groups evaluated were: specialists of breast cancer, family members of Mrs. X, rheumatologists, gynaecologists, cardiologists and physicians in general (Cronbach α = 0.89). Power of control beliefs were assessed with six items presented as follows: "Even if ..., ("Mrs. X has no opinion on HT", "results from the bone mass densitometry are not available", "HT is medicalising menopause", "Mrs. X has not many hot flushes", "Mrs. X has a family history of breast cancer" and "Mrs. X has no risk factors for cardiovascular diseases") I would prescribe HT". These items were assessed on a bipolar 7-point scale ('disagree' to 'agree'). These six items were used to compute a mean composite score for power of control beliefs (Cronbach α = 0.96).

For above variables, the bipolar 7-point scale was rated numerically from -3 to +3. Therefore, a positive score indicated that the physician expressed a positive evaluation of the belief-based construct. Finally, sociodemographics and practice patterns-related variables were assessed with closed-ended questions [37]. Examinations performed variables were assessed with a check list and included elements of the physical examination (for example, breast and pelvic examination) and laboratory (for example, lipid profile) as well as imaging investigations (for example, mammography) that were relevant for a middle-aged woman undergoing a routine periodical medical exam.

### Data analysis

**Figure 2:**
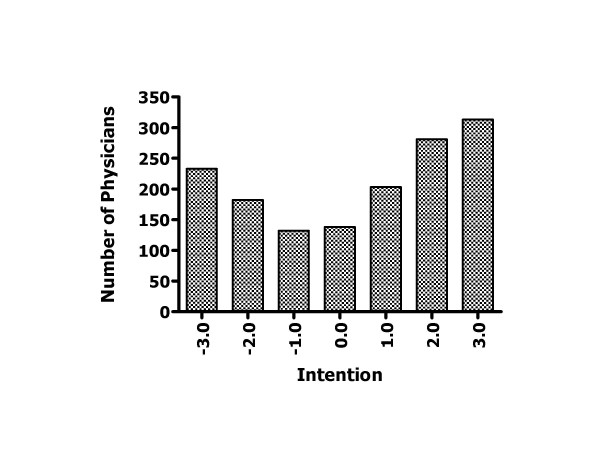
Distribution of the intention to prescribe HT.

Descriptive analyses to assess the distribution of all the explanatory variables were performed. They all showed a near normal to normal distribution. Some elements in the theory of planned behaviour constructs had missing information. If less than one third of the responses were missing for a given construct, then the mean of the other responses within the construct was used to impute these missing answers, otherwise the subject was considered as having too many missing data and was not included in the analysis. The means and SD of psychosocial variables were analyzed by medical specialty and by country using Student's *t*-test with the Bonferronni correction for multiple group comparisons. A U-shaped distribution of the dependant variable that is, the intention to prescribe HT was observed (Figure 2). This type of distribution was not amenable to a transformation. For further analysis, in line with the theory of planned behaviour, the intention to prescribe HT was dichotomized as follows: mean scores of 1 and more were classified as high intention, whereas mean scores of less than 1 were classified as low intention. Multiple logistic regression analysis was used to determine factors associated with high intention.

First, an overall model (all doctors) was produced. Then, doctors were grouped according to their specialty and country of origin. Intention to prescribe HT was first regressed on the direct measure of the main constructs of the theory of planned behaviour (attitude, subjective norm and perceived behavioural control). Second, role belief, moral norm and power of control beliefs were added to the variables retained in the model. Third, attitudinal beliefs and normative beliefs were added. Last, only if they were significant at a p of 0.20 or less in a bivariate analysis with intention to prescribe HT, variables that were external to the initial theoretical model (sociodemographics and practice patterns-related variables) were tested. Adjusted odds ratio and their 95% confidence intervals were computed. All reported *p *values were two-sided. No interaction term was tested. The Statistical Analysis System (SAS Institute, Cary, NC) was used for data analysis. This study was approved by the Ethics Committee of the University Centre where it was conducted.

## Results

### Characteristics of respondents

Overall, 1,085 French doctors returned their questionnaire (response rate = 54%) and 516 doctors from Quebec (response rate = 49%). However, due to missing data, 1011 questionnaires from France (425 GPs and 586 gynaecologists) and 464 questionnaires from Quebec (334 GPs and 130 gynaecologists) were analyzed. A larger proportion of the respondents were gynaecologists in France when compared with respondents in Quebec (58% versus 28%). This difference was due to the different GPs/gynaecologists ratio in the two samples invited to participate in each country: half of the doctors in the France sample were gynaecologists compared with 23% in the Quebec sample. In France, as in Quebec, respondents were significantly more likely to be women (in France 41% of the respondents and 33% of the nonrespondents, *p *< 0.01; in Quebec 37% of the respondents and 31% of the nonrespondents, *p *< 0.05). Characteristics of the participants are presented in Table 1.

**Table 1:** Characteristics of participating doctors by country and medical specialty

	Quebec		France	
	
	General Practitioners n = 334 (%)	Gynaecologists n = 130 (%)	General Practitioners n = 425 (%)	Gynaecologists n = 586 (%)
Women	140 (42%)	35 (27%)	98 (23%)	316 (54%)
Age (years)	41.5 ± 8.6	47.4 ± 10.8	43.7 ± 7.6	45.4 ± 8.3
				
Number of years in practice				
<8 years	82 (25%)	31 (24%)	109 (26%)	107 (18%)
9 – 15 years	92 (27%)	20 (15%)	116 (27%)	236 (40%)
16 – 21 years	90 (27%)	28 (21%)	125 (29%)	124 (21%)
>22 years	70 (21%)	51 (39%)	75 (18%)	119 (21%)
				
Rural place of practice	88 (27%)	8 (6%)	162 (38%)	55 (9%)
Number of patients/day*				
<10	19 (6%)	6 (5%)	15 (4%)	42 (7%)
10 – 20	95 (29%)	20 (15%)	170 (40%)	299 (52%)
20 – 40	191 (58%)	83 (64%)	221 (53%)	224 (39%)
>40	24 (7%)	21 (16%)	14 (3%)	15 (2%)
				
% of menopausal patients in clientele	29.1 ± 25.0	56.0 ± 24.8	21.4 ± 20.2	50.5 ± 27.3
% who use menopausal diagnostic criteria**				
Symptoms	118 (35%)	64 (49%)	174 (41%)	290 (49%)
Hormonal dosages	101 (30%)	23 (18%)	126 (30%)	173 (30%)
Cessation of menses	227 (68%)	78 (60%)	268 (63%)	328 (56%)
Discuss HT with all menopausal patients	122 (38%)	66 (51%)	101 (24%)	355 (61%)
Examinations performed**				
Cytology (PAP smear)	292 (87%)	129 (87%)	363 (85%)	519 (89%)
Mammography	288 (86%)	120 (92%)	381 (90%)	578 (99%)
Breast examination	306 (92%)	127 (98%)	377 (89%)	580 (99%)
Hormonal dosages	113 (34%)	17 (13%)	139 (33%)	152 (26%)
Lipid profile	245 (73%)	59 (45%)	349 (82%)	387 (66%)
Endometrial biopsy	3 (1%)	16 (12%)	11 (3%)	40 (7%)
Bone mass density	30 (9%)	16 (12%)	84 (20%)	77 (13%)
Vaginal ultrasound	1 (0,3%)	4 (3%)	21 (5%)	158 (27%)
Thyroid gland examination	251 (75%)	61 (47%)	187 (44%)	156 (27%)
Pelvic examination	270 (81%)	126 (97%)	307 (72%)	569 (97%)
Speculum	272 (81%)	126 (97%)	276 (65%)	570 (97%)
Blood pressure	308 (92%)	119 (92%)	391 (92%)	569 (97%)
Cardiac auscultation	285 (85%)	29 (22%)	363 (85%)	148 (25%)
Weight	257 (77%)	58 (45%)	341 (80%)	505 (86%)
HT prescription patterns > 70% of cases	106 (34%)	86 (67%)	36 (9%)	309 (55%)

* Total number of doctors within the subgroup might differ from total number of participating doctors because of missing data.

** Categories are not mutually exclusive.

### Means and standard deviation (SD) of the psychosocial variables by medical specialty and by country

Table 2 summarizes the means and standard deviation (SD) of the psychosocial variables. In France, compared with GPs, measures of all the psychosocial variables were higher for gynaecologists (all at *p *< 0.01). In Quebec, compared with GPs, measures of the following psychosocial variables were higher for gynaecologists: intention to prescribe HT (*p *< 0.05) and attitude, subjective norm, perceived behavioural control and moral norm (all at *p *< 0.01). Compared with gynaecologists from Quebec, those from France showed a higher score for attitudinal beliefs (*p *< 0.01). There were no differences between GPs from France and those from Quebec.

**Table 2:** Means and standard deviation (SD) of the psychosocial variables by medical specialty and by country

	Quebec		France	
Psychosocial constructs	General Practitioners n = 334	Gynaecologists n = 130	General Practitioners n = 425	Gynaecologists n = 586
Intention	0.030 ± 2.121	0.642 ± 2.086*	-0.195 ± 2.216	0.556 ± 2.083**
Attitude	0.450 ± 1.456	0.951 ± 1.456**	0.265 ± 1.678	0.847 ± 1.492**
Subjective norm	0.026 ± 1.626	0.594 ± 1.641**	-0.220 ± 1.621	0.316 ± 1.665**
Perceived behavioural control	0.053 ± 1.800	0.740 ± 1.721**	-0.254 ± 1.929	0.560 ± 1.921**
Role belief	0.180 ± 1.809	0.535 ± 1.706	0.102 ± 1.821	0.521 ± 1.729**
Moral norm	0.585 ± 1.771	1.269 ± 1.671**	0.281 ± 2.037	0.963 ± 1.784**
Power of control beliefs	0.195 ± 1.960	0.614 ± 1.974	-0.041 ± 2.143	0.607 ± 2.057**
Behavioural beliefs	1.301 ± 0.975	1.525 ± 0.965	1.374 ± 1.061	1.963 ± 0.819**^¶^
Normative beliefs	0.438 ± 1.396	0.506 ± 1.223	0.287 ± 1.445	0.615 ± 1.270**

Statistical significance given after Bonferroni correction for multiple comparisons:

* p < 0.05 between General practitioners and Gynaecologists for each country

** p < 0.01 between General practitioners and Gynaecologists for each country

^¶ ^p < 0.01 between Gynaecologists between countries

### Psychosocial factors associated with the intention to prescribe HT

The results of the regression analysis are presented in Table 3. In the overall model (all doctors), three psychosocial variables were associated with high intention: power of control beliefs, moral norm and role belief (all at *p *< 0.0001). Since neither the country of origin nor the specialty was found statistically significant in this overall model, and because of the difference in distribution of these factors, separate models were prepared to assess the variables associated with intention to prescribe HT for each of the four combinations. For GPs from Quebec, two psychosocial variables were associated with high intention: power of control beliefs (*p *< 0.0001) and moral norm (*p *< 0.01). Two variables that were external to the initial theoretical model were also retained in the final model: cytology and using hormonal dosage as a menopausal criterion (both at *p *< 0.05). For GPs from France, three psychosocial variables were associated with high intention: power of control beliefs (*p *< 0.0001), role belief (*p *< 0.0001) and perceived behavioural control (*p *< 0.05). One variable that was external to the initial theoretical model was added to the final model: using cessation of menses as a menopausal criterion (*p *< 0.01). For gynaecologists from Quebec (n = 129), two psychosocial variables were associated with high intention: moral norm (*p *< 0.01) and power of control beliefs (*p *< 0.01). No variables that were external to the initial theoretical model were added to this final model. For gynaecologists from France (n = 577), three psychosocial variables were kept in the final model: power of control beliefs (*p *< 0.0001), moral norm (*p *< 0.05), role belief (*p *< 0.05). One variable that was external to the initial theoretical model was added to the final model: performing a lipid profile (*p *< 0.05).

**Table 3:** Psychosocial factors and other variables predicting intention to prescribe HT

	Quebec and France	Quebec		France	
	
	All doctors N = 1472	General Practitioners N = 333	Gynaecologists N = 129	General Practitioners N = 419	Gynaecologists N = 577
	OR (95% CI)	OR (95% CI)	OR (95% CI)	OR (95% CI)	OR (95% CI)

TPB Constructs					
RB	2.08**** (1.61 – 2.69)	-	-	4.60**** (2.17 – 9.73)	1.47* (1.03 – 2.11)
MN	2.38**** (1.70 – 3.34)	3.42** (1.52 – 7.66)	84.58** (3.64 – 4.00)	-	1.75* (1.11 – 2.77)
PBC	-	-	-	1.65* (1.07 – 2.55)	-
Power of control beliefs (POC's)	4.92**** (3.51 – 6.90)	21.92**** (6.86 – 70.05)	15.86** (1.97 – 128.23)	5.80**** (3.05 – 11.03)	6.20**** (3.63 – 10.59)
Other Variables					
Cytology (PAP smear)	-	11.05* (1.01 – 123.08)	-	-	-
Hormonal dosage as menopausal criteria	-	4.36* (1.15 – 16.50)	-	-	-
Cessation of menses as menopausal criteria	-	-	-	4.44*** (1.45 – 13.59)	-
Lipid profile	-	-	-	-	2.64* (1.23 – 2.68)

	Likelihood Ration X^2 ^= 1580; degrees of freedom = 3; p < 0.0001	Likelihood Ration X^2 ^= 384; degrees of freedom = 4; p < 0.0001	Likelihood Ration X^2 ^= 153; degrees of freedom = 2; p < 0.0001	Likelihood Ration X^2 ^= 477; degrees of freedom = 4; p < 0.0001	Likelihood Ration X^2 ^= 584; degrees of freedom = 4; p < 0.0001

Note: Due to incomplete information in some sections of the questionnaire, 996 questionnaires from France (419 general practitioners and 577 gynaecologists) and 462 questionnaires from Quebec (333 general practitioners and 129 gynaecologists) were used in the regression analysis. The differences in the total numbers of subjects in the regressions are due to missing information for some of the explanatory variables in the subgroup models. The outcome variable in the regressions is a dichotomous variable representing high vs. low intention to prescribe.

OR: Odds ratio; 95% CI: 95% Confidence Interval

* p < 0.05, ** p < 0.01, *** p < 0.001, **** p < 0.0001

## Discussion

To our knowledge, this is the first study that adopted the theory of planned behaviour as a theoretical basis for assessing and comparing the psychosocial determinants of GPs and gynaecologists' intention to prescribe HT in two countries, France and in Quebec. To our knowledge, this is also one of the few studies to adopt the theory of planned behaviour for identifying factors that influence doctors' behavioural intention to prescribe a medication in the context of preference-sensitive care.

The results of this study indicated that doctors' intention to prescribe HT varied according to their specialty. In both countries, gynaecologists showed higher intention to prescribe HT than GPs. This is in line with the results reported by Hovi and colleagues who assessed Estonian and Finnish GPs and gynaecologist opinions and prescribing practices in HT [19]. However, this intention did not vary significantly between GPs in France and those in Quebec, nor did it between gynaecologists in France and those in Quebec.

In the overall model (for all physicians in both countries), perceived barriers to prescription of HT was found to be the most important determinant of the intention to prescribe HT. This means that the more confident a doctor was regarding his/her ability to counter the perceived barriers that were assessed in this study, the more likely he/she was to express a high intention to prescribe HT. This is congruent with the observation made previously that a significant proportion of physicians considered menopause to be a serious health problem that needed to be treated [3]. In other words, even in the absence of a clear indication to prescribe HT (for example, severe hot flashes) or a clear indication by a woman that she would like to use HT, at that the time this study was conducted, some physicians had high intention to do so.

Moral norm was found to be the second most important determinant. In this study, moral norm was assessed with three items referring to the physician's personal convictions, sense of guilt and ethics. In the past, moral norm was found to be the strongest predictor of women's intention to adopt HT over a period of one year [32]. However, to our knowledge, the present study is one of the first to document that moral norm is associated with a doctor's prescription behaviour of HT. This means that, in this context, doctors' most profound personal values might overcome those of the women. As reported before by Hoffmann and colleagues, this suggests that doctors might be inclined to promote their own decision [38]. On one hand, this is in line with the literature that points to the contribution of doctors' preferences on the unexplained practice variation observed in the medical community [39]. On the other hand, this is contrary to the growing social trends in regards with shared decision-making [40]. Shared decision-making is grounded in ethics in which a deliberative model of decision-making is advocated [41]. A deliberative model of decision-making recognizes that both protagonists are competent individuals. It refers to the concepts of autonomy and self determination which underline the obligation of health care providers to provide information [41]. Shared decision-making emphasizes the importance of agreement on the treatment option by the patient and the physicians as a "the test of a shared decision" because "mutual acceptance ... remains a necessary prerequisite for shared decision-making" [42]. Shared decision-making rests on the best evidence of the risks and benefits of all the available options and takes into account the establishment of a context in which the values and preferences of the patient are sought and his opinions valued [40]. Therefore, these results suggest a need for interventions that will facilitate the inclusion of the specific characteristics and values of the patient in the decision. In the overall model, the intention to prescribe HT was also under the influence of role belief. This means that intention to prescribe HT was determined by a sense of belonging to a specific group and how one ought to act accordingly. This is different from the influence of subjective norm, which refers to perceived social pressure to perform a specific behaviour. In other words, doctors' intention to prescribe HT was dependent on what they believe someone like them should do. Doctors attach a high value to their clinical autonomy, which is viewed by certain critics as a major barrier to changing doctors' behaviour [43]. The positive influence of role belief in predicting doctors' behaviour had been reported before in the area of technology of information [24]. It appears that in the context of HT prescription, doctors might not be influenced by what others would want them to do (including a specific patient), but rather by what they believe a doctor like themselves ought to do.

When specific regression models were built by medical specialty and country, power of control beliefs was kept in all models. Moral norm was kept in three out of the four models: GPs in Quebec and gynaecologists in France and in Quebec. Role belief was kept in both models from France: GPs and gynaecologists, but not in the models of doctors from Quebec (GPs and gynaecologists). This suggests that, notwithstanding the medical specialty, there might be differences between doctors from France and those from Quebec regarding this particular behaviour.

In three out of the four models, variables that were external to the initial theoretical model made a statistically significant contribution. All of these variables pertained to diagnostic testing. For example, in each of the regression models pertaining to GPs' behavioural intention, variables associated with diagnosing menopause were found. This suggests that GPs in Quebec and in France might need reassurance in the final objective diagnosis of menopause before embarking on a prescription of HT.

Interestingly, attitude and attitudinal beliefs were not retained in any of the regression models. This means that the intention of doctors to prescribe HT was not under the influence of those doctors' personal evaluation of this action (for example, the consequences of prescribing HT on the woman's health outcomes). This suggests that, when controlled for other psychosocial variables, the prescription of HT was not under the influence of what doctors believe HT would have had as potential benefits or risks to this woman.

Another interesting finding of the present study was the U-shaped distribution of the doctors' intention to prescribe HT. Indeed, given the recommendation regarding HT by some of the professional societies at the time this study was conducted [44-47], one could have expected a skewed distribution towards a more positive physicians' intention to prescribe HT. This U-shaped distribution of the doctors' intention to prescribe HT is in line with the results of a previous study published in 1986, long before HT was thought to be benefiting the cardiovascular system. Interestingly, in this study published in 1986, doctors were shown to act decisively when facing decisions about HT [14]. Facing twelve clinical vignettes, most of the fifty doctors enrolled in this study tended to act decisively (prescribe HT or not) although the decision analysis model had recommended a "toss-up" strategy (uncertain decision) 60% of the time. The authors concluded that "doctors ought to carefully re-examine their practice patterns in light of their beliefs and opinions." [14]. This conclusion would still hold true two decades later. It suggests that it is possible that physicians' intention to prescribe HT and its determinants could be stable overtime.

This U-shaped distribution of the doctors' behavioural intention regarding HT was in sharp contrast with the results from two other studies that applied the theory of planned behaviour to predict middle-aged women intention to use HT at menopause in Québec. These two studies were conducted in the same period as the present study was. In both these studies, the distribution of the intention to use HT was normal. In other words, while most women appeared to be undecided about adopting HT, physicians from the same geographic area appeared to be decided about either prescribing HT or not. Again, these results emphasise the need for future interventions that will facilitate the active participation of women in the decision-making leading to a prescription of HT.

### Strengths and limitations of the study results

A major strength of this study is its use of theory to improve understanding of the decision-making of doctors leading to a prescription of HT. Therefore, the use of an existing theoretical framework helped standardize the presentation of the many factors that are likely to influence this prescription behaviour [48]. It has the potential to: 1) facilitate the comparison between similar studies, 2) make it possible to carry out a systematic review in this area, and 3) contribute to the elaboration of a theoretical base for understanding the decision-making leading to a prescription behaviour. Other researchers might want to follow this process.

In spite of these strengths, this study has limitations. First, given that this study was conducted before the results of the Women's Health Initiative trial were publicized [1], we can not guarantee that its results would still be applicable. The decline in postmenopausal HT use was most marked for standard-dose of the estrogenic agent used in the Women's Health Initiative trial [9]. However, contrary to this trend, prescription for lower-dose formulations increased modestly [9]. Moreover, in France, results from the Women's Health Initiative trial have been criticized by French professional societies based on the fact that treatments used were different in France – mainly transdermal estrogens – and that French postmenopausal women were at lower vascular risk than those of the Women's Health Initiative trial [49]. In United States, studies of women younger than those enrolled in this trial and lower HT doses with intermediate endpoints are beginning [50]. Therefore, given the clinical vignette that was used in this study (there was no explicit dosage and formulae of HT), we believe that this study results might still be applicable to the current situation.

Second, doctors were asked to indicate their intention to prescribe HT to a specific but hypothetical woman. The clinical vignette that was used was succinct and did not provide all the clinical information that some doctors might have desired. However, a large proportion of doctors who participated in this study expressed a specific intention to prescribe HT or not, perhaps indicating that they felt they had enough information to make a decision. Moreover, clinical vignettes are known to be a valid and comprehensive method to asses the process of care provided in actual clinical practice [51]. Therefore, we believe our results can improve our understanding of the prescription of HT in this specific clinical situation.

Last, although this study was designed to draw a representative sample of GPs and gynaecologists from each country, we can not guarantee that our results apply to the whole population of GPs and gynaecologists from which our sample was drawn. The response rate that was obtained in both countries compared well with other studies that have applied the theory of planned behaviour to study doctors' behaviour [24,25,52]. Nonetheless, this response rate as well as the ratio of GPs and gynaecologists limits the inference of our results to the whole population of doctors. However, it does not limit the inference of the overall regression model for this group of respondents. Thus, the results of this study provide a valuable contribution to the theory base of doctors' prescription behaviour.

## Conclusion

Despite its limitations, the present study still has clinical and research implications. Geographic variations in health care delivery remain a challenge [39]. This study provides empirical data on the significant contribution of moral norm in the decision-making leading to a prescription of HT by doctors and possibly in other contexts of preference-sensitive care [39]. This is preoccupying because it is contrary to on-going efforts to incorporate the best available scientific evidences as well as the patients' views in health [53], one of the cornerstones of improving the quality of health care [54]. Therefore, it would be important in this type of clinical situation to provide guidance to doctors on how to support active participation of patients in the decision-making process.

This study should be useful to those interested in the application of social cognitive models in studying doctors' behaviour. Given the lack of theorization in the area of health care professional practice [20,55], this study results improve the knowledge base in translation of evidence as well as the knowledge base of physicians' decision-making in context of preference-sensitive care [39]. It provides insight about the contribution of moral norm and role beliefs above variables provided by the theory of planned behaviour to explain doctors' behaviour and perhaps, variation in health care delivery. In future studies applying the theory of planned behaviour, these constructs should be tested with other type of doctors' behaviours. This study should also be useful in improving our understanding of cross-cultural variations in medical practices by focusing on theory-based psychosocial variables. Lastly, this study points to possible pathways through which a given implementation strategy is likely to be effective in modifying doctors' prescription of HT. Researchers interested in elaborating interventions to modify this prescription behaviour could use this study results accordingly.

## Competing interests

The author(s) declare that they have no competing interests.

## Authors' contributions

France Legare, Gaston Godin, Sylvie Dodin and Virginie Ringa designed the study with assistance from Joanna Norton. Data were collected and managed by Lucile Turcot and France Legare. Data were analyzed by France Legare, Gaston Godin and Lucile Turcot. France Legare wrote the report. All authors contributed to the revision of various drafts and approved the final version of the submitted manuscript.

## Pre-publication history

The pre-publication history for this paper can be accessed here:


